# Primary Apocrine Adenocarcinoma of the Vulva: A Report of a Rare Case

**DOI:** 10.7759/cureus.105277

**Published:** 2026-03-15

**Authors:** Marcus Yoakam, Jesse Friday, Andrea Weitoschova, Kalyn Borger, Drew Windsor, Brett Dunbar, Victoria Shirley

**Affiliations:** 1 College of Osteopathic Medicine, Kansas City University, Joplin, USA; 2 Department of General Surgery, Mercy Hospital Pittsburg, Pittsburg, USA; 3 Department of Obstetrics and Gynecology, Mercy Hospital Pittsburg, Pittsburg, USA

**Keywords:** apocrine adenocarcinoma, rare cancer of female genital tract, rare case, vulvar neoplasm, wide local excision (wle)

## Abstract

Primary apocrine adenocarcinoma of the vulva is a rare and aggressive malignancy that typically presents as an irregular vulvar lesion with associated dermatologic changes. Diagnosis can be challenging due to significant clinical and histopathologic overlap with other vulvar adenocarcinomas as well as metastatic disease. We present the case of a 63-year-old postmenopausal woman diagnosed with primary apocrine adenocarcinoma of the vulva. The patient’s prior history of breast cancer created a diagnostic dilemma, as it was initially unclear whether the lesion represented metastatic breast carcinoma or a primary vulvar malignancy. Histopathologic evaluation with immunohistochemical analysis demonstrated strong androgen receptor positivity, negative estrogen receptor and progesterone receptor staining, strong GCDFP-15 positivity, and strong CK7 positivity. This profile supported the diagnosis of primary vulvar apocrine adenocarcinoma, allowing for appropriate surgical management and successful excision of the lesion. This case highlights the diagnostic complexity of vulvar adenocarcinomas and underscores the importance of systematic clinicopathologic evaluation, particularly in patients with a history of malignancy.

## Introduction

Primary apocrine adenocarcinoma of the vulva is an extremely rare malignancy that primarily affects postmenopausal women [1]. Clinically, it often presents as an irregular vulvar mass or lesion with possible skin changes (e.g., ulceration and eczematous appearance) and may be associated with local lymphatic metastases [2,3]. Because the vulva contains multiple glandular elements, differential diagnoses include Bartholin gland adenocarcinoma, mammary-like (cutaneous) adenocarcinoma, extramammary Paget’s disease with underlying adnexal carcinoma, and other adnexal neoplasms (e.g., eccrine carcinoma). Moreover, metastatic adenocarcinoma, particularly from the breast or gastrointestinal tract, must be excluded, especially in patients with prior malignancy [4,5].

The diagnosis is challenging due to overlapping histological features with other vulvar adenocarcinomas and requires a combination of thorough clinical examination, histopathological evaluation, and immunohistochemical profiling. Histopathology often shows glandular structures with apocrine differentiation, and immunohistochemistry typically reveals positivity for CK7, EMA, GCDFP-15, and GATA3, while negative markers such as CK20 and CDX2 help exclude colorectal or other non-apocrine primaries. Correlation with clinical history and additional markers, such as mammaglobin in breast cancer, is essential to distinguish primary vulvar disease from metastatic tumors [6-8]. The standard management involves surgical excision, with the choice between wide local excision and radical vulvectomy determined by tumor size and extent, along with additional lymphadenectomy if there is nodal involvement. Adjuvant radiotherapy and chemotherapy may be considered in cases with positive margins or advanced disease [9,10]. Long-term, individualized follow-up is essential due to the risk of recurrence.

Primary apocrine adenocarcinoma of the vulva is a rare and diagnostically challenging malignancy within gynecologic oncology. Its clinical importance stems from its aggressive behavior, potential for local and distant metastasis, and the complexity of distinguishing it from other vulvar adenocarcinomas and metastatic lesions. The rarity of the disease has resulted in a limited evidence base, with most knowledge derived from case reports and small series, underscoring the need for improved understanding of its epidemiology, diagnosis, and management [1,11].

## Case presentation

A 63-year-old postmenopausal female presented to the clinic for a preoperative evaluation prior to excision of a vulvar lesion. The patient’s past medical history was significant for a previous low-grade squamous intraepithelial lesion, a positive HPV test, and a prior abnormal Pap smear with HPV-positive/ASCUS findings. Additionally, she had a history of an estrogen receptor (ER) and progesterone receptor (PR) positive primary malignant neoplasm in the central portion of the breast. The patient subsequently underwent a right mastectomy, followed by six cycles of adjuvant cyclophosphamide, doxorubicin, and fluorouracil chemotherapy. She also had a notable history of premature menopause and obesity.

On examination, the patient presented with a raised, nodular lesion on the left vulva. She reported abnormal vaginal bleeding but denied other significant symptoms. Musculoskeletal and neurologic examinations were unremarkable. Previous biopsies of the lesion suggested lichen sclerosis; however, this diagnosis was deemed unlikely due to the raised and indurated nature of the lesion. The patient subsequently underwent wide local excision of the vulvar lesion in January 2024, with negative margins confirmed by the surgical pathology report. She experienced no postoperative complications, and the incision site healed well without signs of infection or other issues.

**Figure 1:**
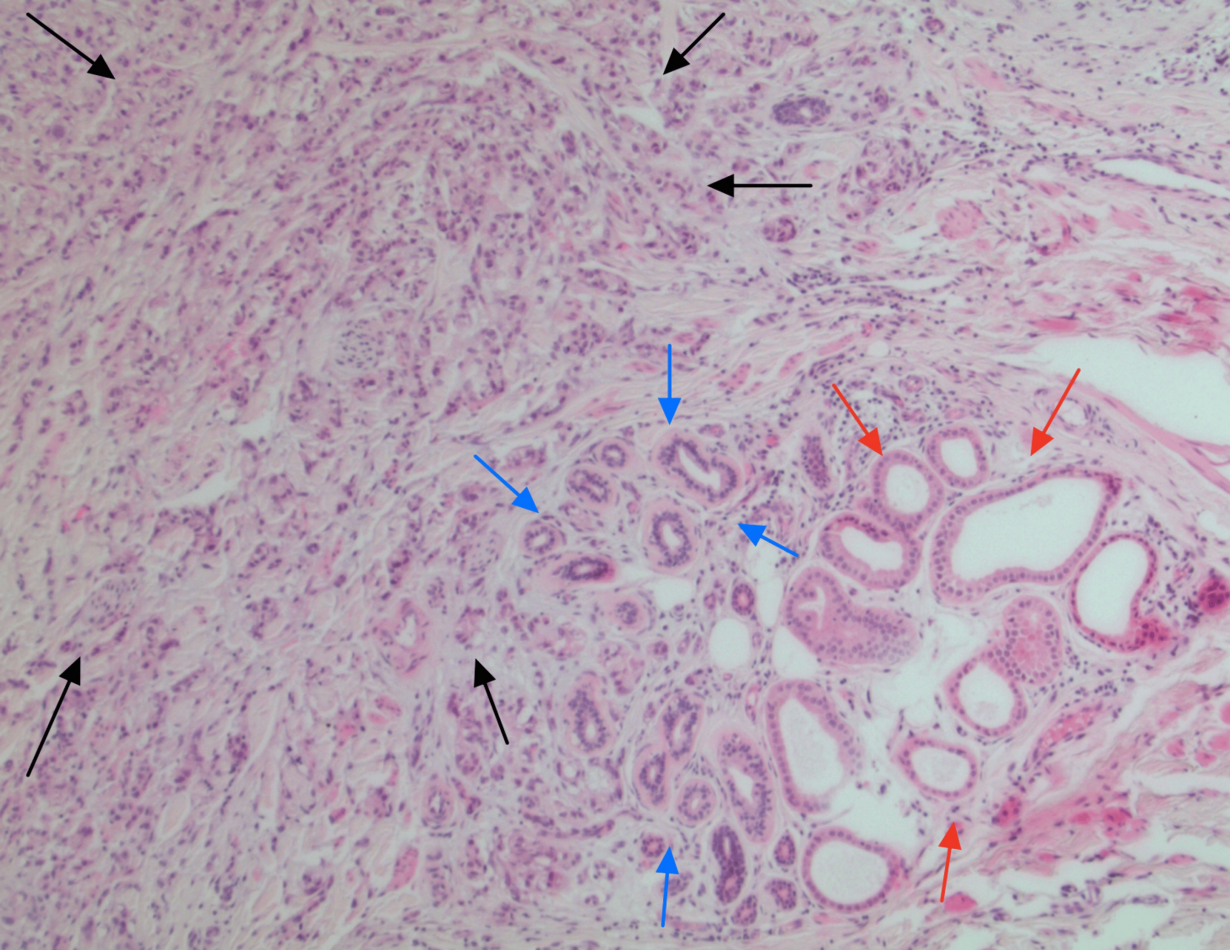
H&E tumor and native apocrine (40x magnification) Native apocrine glands are located in the lower right of the image (red arrows). Eccrine glands are located to the left of the apocrine glands (blue arrows). The invasive apocrine carcinoma is visible on the left side of the image (black arrows).

In this patient case, a lesion measuring 3.9 cm by 2.7 cm with a depth of 2.4 cm was excised from the left vulva. The pathologic description noted a 2.6 cm by 2.2 cm raised, nodular lesion on the epidermal surface. Sectioning through the mass revealed a firm, white-tan cut surface with ill-defined edges. The mass invaded to a depth of 0.5 cm and was present 0.3 cm from the nearest skin margin at the 3 o’clock position. A representative H&E-stained section is shown in Figure 1, demonstrating the tumor’s underlying histomorphology among native apocrine glands.

**Figure 2:**
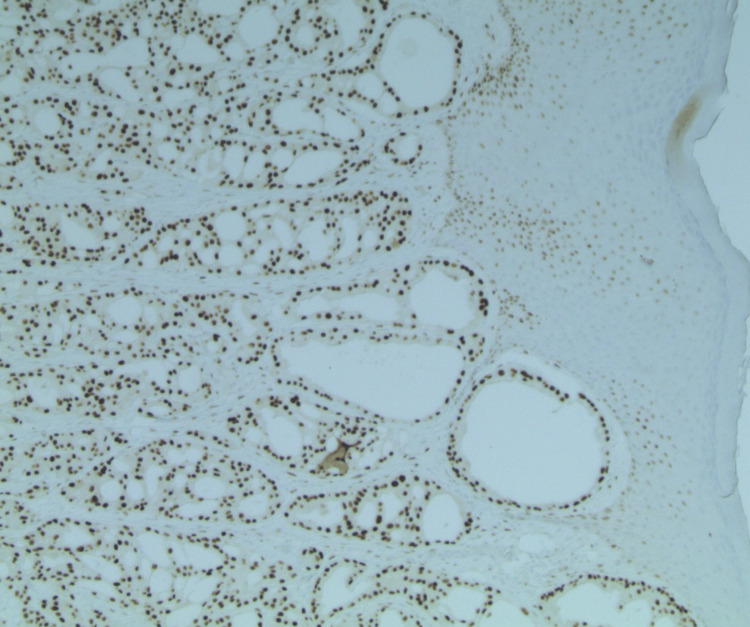
Strongly positive AR stain tumor (100x magnification) AR, androgen receptor

**Figure 3:**
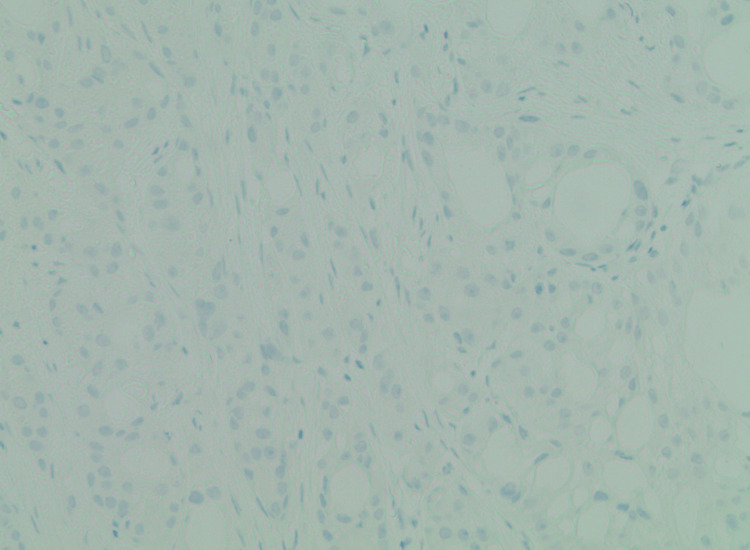
Negative PR stain tumor (100x magnification) PR, progesterone receptor

**Figure 4:**
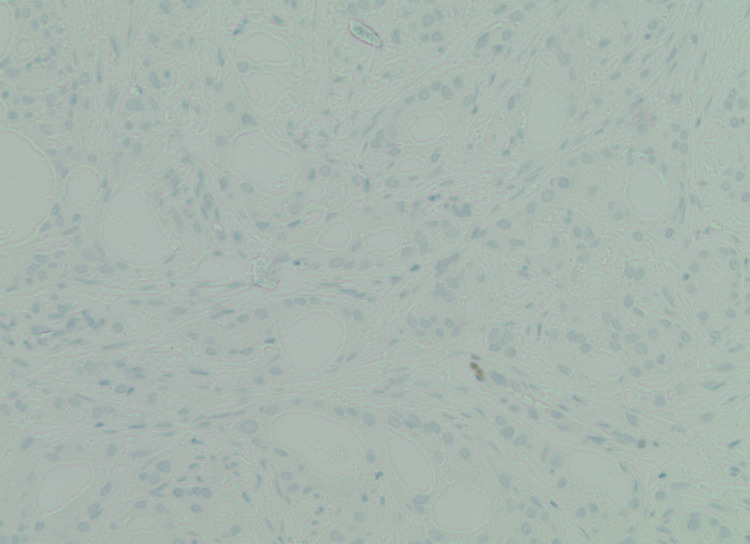
Negative ER stain tumor (100x magnification) ER, estrogen receptor

**Figure 5:**
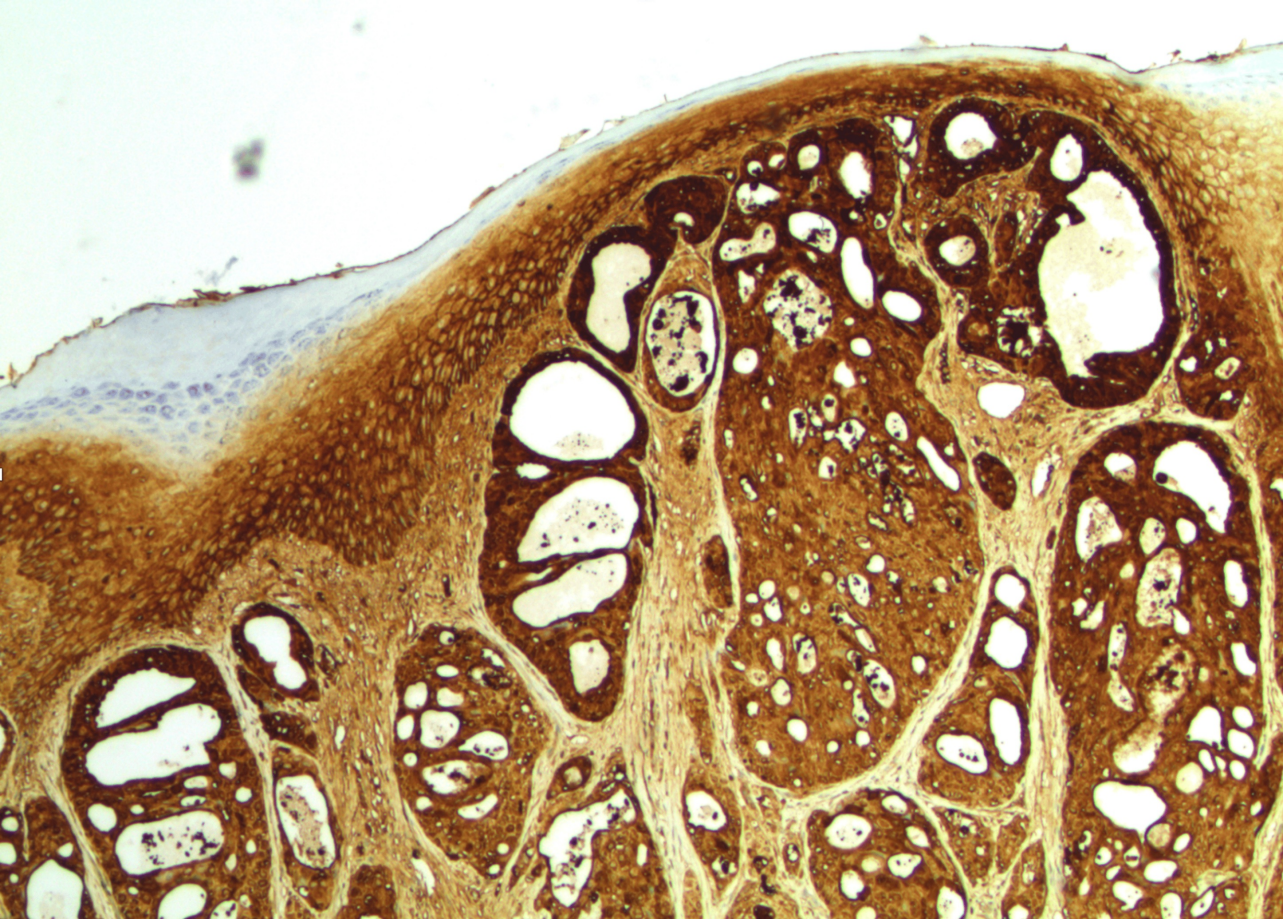
Strongly positive GCDFP-15 stain tumor (40x magnification)

Immunohistochemical staining demonstrated strong androgen receptor (AR) positivity (Figure 2), negative ER and PR staining (Figure 3, Figure 4), strong positivity for GCDFP-15 (Figure 5), and strong CK7 positivity.

Taken together, these findings were most consistent with a diagnosis of apocrine carcinoma rather than metastatic breast carcinoma. Following consultation between two pathologists, the final submitted diagnosis was primary apocrine adenocarcinoma of the vulva.

A follow-up PET scan in February 2024 showed no areas of increased metabolic activity, indicating no evidence of metastatic disease at that time. The patient reported no new symptoms or postoperative complications.

## Discussion

The most common primary differential diagnosis is metastatic breast carcinoma to the vulva. Cutaneous apocrine carcinomas are more likely to be adipophilin-negative or only focally positive, ER/PR-negative, and may show strong CK5/6 expression, whereas mammary apocrine carcinomas typically demonstrate strong, diffuse adipophilin staining [12]. Clinical correlation and exclusion of a breast primary are essential for a definitive diagnosis.

The immunohistochemical profile of apocrine carcinomas is defined by a characteristic constellation of markers supporting apocrine differentiation. GCDFP-15 is consistently positive and is considered a reliable marker, showing cytoplasmic staining in approximately 75-97% of cases [8,13]. CK7 expression is also consistently observed in apocrine carcinomas, while p63 is typically negative, a finding that helps exclude squamous differentiation. Additional supportive immunohistochemical features include strong AR positivity, which is closely correlated with pure apocrine carcinoma morphology and is regarded as a hallmark of apocrine differentiation, particularly when combined with negative estrogen and progesterone receptor status [14,15]. Consistent with this profile, apocrine carcinomas generally lack ER and PR expression [12,14,16].

Historically, the pathohistology of primary adenocarcinoma of the vulva has been poorly understood due to its extreme rarity. This specific subtype of vulvar malignancy accounts for less than 0.1% of all cases, with the majority being of squamous cell carcinoma etiology. Debate regarding the exact origin of this adenocarcinoma is split between the histogenesis of native apocrine sweat glands in the vulvar region and anogenital mammary-like glands found in the region, which demonstrate cell markers similar to breast tissue [17]. The subject of this case report is unique due to a prior history of breast cancer, suggesting a potential link to the mammary-like gland hypothesis. While these lesions were historically postulated as ectopic breast tissue, current evidence points to malignant transformation of anogenital skin, likely derived from eccrine or apocrine glands entirely distinct from breast tissue [18,19].

In this patient, the presence of a vulvar adenocarcinoma in the setting of previous breast malignancy created a diagnostic dilemma. Systematic evaluation, including detailed histopathologic assessment and targeted immunohistochemical studies, was essential to distinguish a primary vulvar apocrine adenocarcinoma from metastatic disease. The immunohistochemical profile ultimately supported a primary vulvar origin, allowing for appropriate management. Following accurate diagnosis, the lesion was successfully treated with surgical excision, with no evidence of hypermetabolic activity on a follow-up PET scan. This case highlights the importance of maintaining a broad differential diagnosis when evaluating vulvar adenocarcinomas, particularly in patients with a history of malignancy, and underscores the critical role of immunohistochemistry in resolving complex diagnostic scenarios.

## Conclusions

Vulvar adenocarcinoma generally carries a favorable prognosis when identified early, before involvement of regional lymph nodes. In contrast, metastasis to the superficial inguinal lymph nodes, which drain the vulva, including the labia majora and minora, is associated with significantly worse outcomes. Prompt recognition and timely intervention are therefore critical to limiting disease progression and improving patient prognosis.

In this case report, accurate diagnosis required careful clinicopathologic correlation and immunohistochemical evaluation to distinguish a rare primary vulvar apocrine adenocarcinoma from potential metastatic disease in a patient with a history of breast cancer. This systematic diagnostic approach allowed for appropriate surgical management with successful excision of the lesion.
